# Phenotypic Clustering of Beta-Thalassemia Intermedia Patients Using Cardiovascular Magnetic Resonance

**DOI:** 10.3390/jcm12216706

**Published:** 2023-10-24

**Authors:** Antonella Meloni, Michela Parravano, Laura Pistoia, Alberto Cossu, Emanuele Grassedonio, Stefania Renne, Priscilla Fina, Anna Spasiano, Alessandra Salvo, Sergio Bagnato, Calogera Gerardi, Zelia Borsellino, Filippo Cademartiri, Vincenzo Positano

**Affiliations:** 1 Department of Radiology, Fondazione G. Monasterio CNR-Regione Toscana, 56124 Pisa, PI, Italy; laura.pistoia@ftgm.it (L.P.); fcademartiri@ftgm.it (F.C.); positano@ftgm.it (V.P.); 2 Unità Operativa Complessa Bioingegneria, Fondazione G. Monasterio CNR-Regione Toscana, 56124 Pisa, PI, Italy; michela.parravano97@gmail.com; 3 Dipartimento di Ingegneria dell’Informazione, Università degli Studi di Pisa, 56122 Pisa, PI, Italy; 4 Unità Operativa Complessa Ricerca Clinica, Fondazione G. Monasterio CNR-Regione Toscana, 56124 Pisa, PI, Italy; 5 Unità Operativa Radiologia Universitaria, Azienda Ospedaliero-Universitaria “S. Anna”, 44124 Cona, FE, Italy; csslrt@unife.it; 6 Sezione di Scienze Radiologiche, Dipartimento di Biopatologia e Biotecnologie Mediche, Policlinico “Paolo Giaccone”, 90127 Palermo, PA, Italy; egrassedonio@gmail.com; 7 Struttura Complessa di Cardioradiologia-UTIC, Presidio Ospedaliero “Giovanni Paolo II”, 88046 Lamezia Terme, CZ, Italy; stefania.renne@virgilio.it; 8 Unità Operativa Complessa Diagnostica per Immagini, Ospedale “Sandro Pertini”, 00157 Roma, RM, Italy; priscilla.fina@gmail.com; 9 Unità Operativa Semplice Dipartimentale Malattie Rare del Globulo Rosso, Azienda Ospedaliera di Rilievo Nazionale “A. Cardarelli”, 80131 Napoli, NA, Italy; spasiano.anna@tiscali.it; 10 Unità Operativa Semplice Talassemia, Presidio Ospedaliero “Umberto I”, 96100 Siracusa, SR, Italy; gaetasalvo@gmail.com; 11 Ematologia Microcitemia, Ospedale San Giovanni di Dio—ASP Crotone, 88900 Crotone, KR, Italy; krthal@libero.it; 12 Unità Operativa Semplice Dipartimentale di Talassemia, Presidio Ospedaliero “Giovanni Paolo II”—Distretto AG2 di Sciacca, 92019 Sciacca, AG, Italy; gerardicalogera@gmail.com; 13 Unità Operativa Complessa Ematologia con Talassemia, ARNAS Civico “Benfratelli-Di Cristina”, 90134 Palermo, PA, Italy; zelia.borsellino@arnascivico.it

**Keywords:** clustering, phenomapping, cardiovascular magnetic resonance imaging, thalassemia intermedia

## Abstract

We employed an unsupervised clustering method that integrated demographic, clinical, and cardiac magnetic resonance (CMR) data to identify distinct phenogroups (PGs) of patients with beta-thalassemia intermedia (β-TI). We considered 138 β-TI patients consecutively enrolled in the Myocardial Iron Overload in Thalassemia (MIOT) Network who underwent MR for the quantification of hepatic and cardiac iron overload (T2* technique), the assessment of biventricular size and function and atrial dimensions (cine images), and the detection of replacement myocardial fibrosis (late gadolinium enhancement technique). Three mutually exclusive phenogroups were identified based on unsupervised hierarchical clustering of principal components: PG1, women; PG2, patients with replacement myocardial fibrosis, increased biventricular volumes and masses, and lower left ventricular ejection fraction; and PG3, men without replacement myocardial fibrosis, but with increased biventricular volumes and masses and lower left ventricular ejection fraction. The hematochemical parameters and the hepatic and cardiac iron levels did not contribute to the PG definition. PG2 exhibited a significantly higher risk of future cardiovascular events (heart failure, arrhythmias, and pulmonary hypertension) than PG1 (hazard ratio-HR = 10.5; *p* = 0.027) and PG3 (HR = 9.0; *p* = 0.038). Clustering emerged as a useful tool for risk stratification in TI, enabling the identification of three phenogroups with distinct clinical and prognostic characteristics.

## 1. Introduction

Beta-thalassemias are inherited disorders determined by a decrease (β+) or absence (β0) of the production of β-globin chains [1,2]. The spectrum of beta-thalassemias ranges from thalassemia minor, which presents as mild hypochromic microcytic anemia without significant clinical symptoms, to beta-thalassemia major (β-TM), which is characterized by severe anemia starting in early childhood and requiring lifelong regular blood transfusions [3]. Beta-thalassemia intermedia (β-TI) falls between these two extremes, with manifestations that are less severe than those of β-TM, but more significant than those of thalassemia minor [4]. β-TI is categorized as one of the non-transfusion-dependent thalassemias (NTDTs), along with mild/moderate HbE/β-thalassemia and α-thalassemia intermedia. NTDT refers to thalassemic conditions in which regular transfusions are unnecessary for the patient’s survival [5]. However, certain β-TI patients may necessitate transfusions during specific circumstances, such as pregnancy, acute infections, and surgery, or may require regular blood transfusions at some stage of their lives to ensure proper growth, prevent complications, or manage existing ones [6,7,8].

In β-TI, the clinical manifestations are heterogeneous and arise from three main underlying factors: ineffective erythropoiesis, chronic anemia, and iron overload. The ineffective production of red blood cells and the presence of chronic anemia result in the suppression of hepcidin, a hormone involved in regulating iron levels [9,10]. This reduction in the hepcidin levels causes an increase in iron absorption from the gastrointestinal tract and prompts the release of previously recycled iron from the reticuloendothelial system [11]. In β-TI, the liver is the main site for iron accumulation, while the heart is relatively spared [12,13,14,15,16,17]. Chronic anemia triggers a series of compensatory mechanisms within the cardiovascular system. Initially, the body responds to hypoxia by increasing cardiac output through various adaptations, including an elevation in heart rate and an increase in stroke volume [18,19]. To sustain the increased workload, the heart undergoes structural changes, including the enlargement of its chambers [20]. Since this remodeling can lead to decreased cardiac function and heart failure over time, regular cardiac monitoring, allowing for prompt intervention and therapy, may help to slow the progression of early stage disease and avoid the onset of overt cardiovascular disease.

Cardiac magnetic resonance (CMR) imaging provides a valuable means to assess and understand the physiological and pathological aspects of the heart. CMR is considered the gold standard for the quantification of biventricular volumes, masses, and functions due to its exceptional accuracy and reproducibility [21,22]. Moreover, CMR is the reference standard for the non-invasive quantification of tissue iron overload by the T2* technique [23,24,25] and allows the non-invasive detection of myocardial fibrosis [22,26,27].

The interpretation and integration of CMR, demographic, and clinical parameters can be complex and challenging, often requiring expert knowledge and experience. Machine learning algorithms provide a means to uncover hidden patterns within complex and heterogeneous datasets [28,29,30]. Supervised learning involves training a model to classify or predict specific outputs or outcomes based on labeled training data. Conversely, unsupervised learning algorithms work by exploring the intrinsic characteristics and organization of the data themselves to discover hidden insights, detect anomalies, or identify similarities or patterns between variables. Common unsupervised learning methods include clustering algorithms that classify data points into distinct groups or categories based on their intrinsic characteristics or proximity to one another [31,32]. Cluster analysis has been successfully applied in healthcare, allowing the identification of phenogroups or clusters with specific clinical and prognostic profiles in different disease settings [33,34,35,36,37,38,39].

The aims of this multicenter study were: (1) to demonstrate the ability of an unsupervised clustering approach to identify phenogroups among patients with β-TI using demographic, clinical, and CMR data; (2) to determine the clinical implications of the detected phenogroups by discovering their association with distinct profiles in the baseline characteristics; and (3) to assess the prognostic value of the detected phenogroups by comparing the cardiovascular outcomes.

## 2. Materials and Methods

### 2.1. Patient Population

We considered 342 β-TI patients (mean age: 38.4 ± 11.7 years; 50.9% females) consecutively enrolled within the Myocardial Iron Overload in Thalassemia (MIOT) Network. The MIOT Network was an Italian Network of 70 thalassemia centers and 10 magnetic resonance imaging (MRI) sites adopting a standardized and homogeneous MRI protocol [40,41]. All patients underwent their first MRI examination between April 2006 and May 2014. According to the study’s protocol, patients underwent regular MRI follow-up examinations every 18 ± 3 months. All the MIOT centers were interconnected through a shared web-based database, where a detailed clinical history of the patients was recorded [42]. The clinical and instrumental data were updated in correspondence to each new MRI scan.

The study received approval from the institutional ethics committee and was conducted in compliance with the principles stated in the Declaration of Helsinki. Informed consent was obtained from all patients.

### 2.2. Patient Follow-Up and Outcomes

A comprehensive follow-up was conducted for all patients until the end of the MIOT project (November 2015). For each patient, a case report form detailing cardiovascular outcomes between the last available MRI and November 2015 was completed by the caring hematologist.

The following cardiovascular events were considered: heart failure (HF), arrhythmias, and pulmonary hypertension (PH). HF diagnosis was performed by clinicians based on symptoms, signs, and instrumental findings according to the American Heart Association (AHA)/American College of Cardiology (ACC) guidelines [43]. Arrhythmias were diagnosed if documented by electrocardiogram (ECG) or 24 h Holter ECG and if specific medications were required. The classification of arrhythmias was carried out according to the AHA/ACC guidelines [44]. PH was diagnosed if the trans-tricuspidal velocity jet measured by echocardiography exceeded 3.2 m/s [45]. If a patient had more than one complication, only the first one was taken into account.

### 2.3. Magnetic Resonance Imaging

MRI examinations were conducted using 1.5 T scanners from three main vendors (GE Healthcare, Milwaukee, WI, USA; Philips, Best, The Netherlands; and Siemens Healthineers, Erlangen, Germany) equipped with phased-array coils. Breath-holding end-expiration and ECG gating were employed for signal reception.

To assess iron overload, T2* gradient-echo multiecho sequences were used. A single axial mid-hepatic slice [40] and three parallel short-axis views (basal, medium, and apical) of the left ventricle (LV) were acquired [41]. Analysis of the T2* images was carried out using custom-written, previously validated software (HIPPOMIOT^®^,Version 1.0, Consiglio Nazionale delle Ricerche and Fondazione Toscana Gabriele Monasterio, Pisa, Italy) [46]. The liver T2* value was measured within a standard-sized region of interest (ROI), carefully selected to be devoid of blood vessels and distant from areas more susceptible to artifacts caused by magnetic susceptibility [40,47]. The conversion of liver T2* into liver iron concentration (LIC) values was performed by employing the calibration curve introduced by Wood et al. [48]. The software provided T2* values for all 16 segments of the LV following the standard AHA/ACC model [49]. To account for susceptibility and geometric artifacts, an appropriate correction map was applied [46]. The global heart T2* value was derived from the average of the 16 segmental T2* values.

To quantify biventricular function parameters, steady-state free procession (SSFP) end-expiration breath-hold cines were acquired in contiguous short-axis slices (slice thickness 8 mm, without gap) from the atrioventricular ring to the apex. The analysis of the images followed a standard procedure conducted by experienced observers [50]. The process involved manual contouring of the epicardial and endocardial borders in relevant slices during the end-diastolic and end-systolic phases. The interventricular septum was considered part of the LV. Additionally, the papillary muscles and trabeculations within the LV and right ventricular (RV) cavities were included in the respective cavity volumes. The end-diastolic volume (EDV) and end-systolic volume (ESV) were calculated without making geometric assumptions. The ejection fraction (EF) was determined by dividing the stroke volume (difference between EDV and ESV) by the EDV. The wall mass was obtained by multiplying the volume of the myocardium by its specific weight, which was assumed to be 1.05 g/cm^3^. The assessment of the RV mass was centralized in the coordinating center of the MIOT Network and performed by a single experienced operator. The left and right atrial areas were assessed from the four-chamber view projection during the ventricular end-systolic phase. To account for individual variations, the biventricular volumes, masses, and atrial areas were indexed to the body surface area (BSA).

To detect the presence of focal/replacement myocardial fibrosis, late gadolinium enhancement (LGE) images were obtained 10–18 min after the intravenous administration of a standard dose (0.2 mmol/kg) of Gadobutrol (Gadovist^®^; Bayer Schering Pharma; Berlin, Germany) using a fast gradient-echo inversion recovery T1 weighted sequence. Short-axis and vertical, horizontal, and oblique long-axis views were acquired. LGE images were not acquired in patients with a glomerular filtration rate inferior to 30 mL/min/1.73 m^2^, nor in patients who declined the administration of the contrast medium. The presence of LGE was determined based on visual assessment in at least two different views [51].

### 2.4. Cluster Analysis

The cluster analysis was performed using the free software R (version 4.3.1).

A total of 16 clinical and CMR variables were selected for the clustering model and the determination of phenogroups. There were three categorical variables (sex, presence of regular transfusions, and replacement myocardial fibrosis) and 13 continuous variables (age, mean serum hemoglobin, mean serum ferritin, MRI LIC, global heart T2* values, LV and RV end-diastolic volume index, LV and RV mass index, LV and RV EF, and left and right atrial area index).

### 2.5. Data Pre-Processing

The raw clinical data were pre-elaborated before the application of clustering algorithms.

To handle the missing data, the complete case analysis strategy was adopted: patients with missing data on any input variable were excluded from the analysis.

To ensure that variables with different ranges contributed equally to the clustering process, normalization of continuous variables was performed. Z-score normalization (or standardization), scaling the data by the mean and standard deviation, was chosen.

### 2.6. Evaluation of Clustering Tendency

The Hopkins statistic was computed by the Hopkins function (Hopkins 1.0 package) to determine if the observations within the dataset were clusterizable [52]. The Hopkins statistic measures the probability that a given data set is created by a uniform data distribution. The Hopkins statistic score is between 0 and 1, with a value of >0.5 indicating that the dataset is clusterizable.

### 2.7. Definition of Phenogroups

Phenogroups were defined using an unsupervised hierarchical clustering on principal components (HCPC) approach, performed by the functions MFA and HCPC of the FactoMineR 2.8 package.

The algorithm consisted of two steps. First, a multiple-factor analysis (MFA) was conducted. MFA is an extension of principal component analysis (PCA), which is able to handle both continuous and categorical variables [53]. PCA is a technique for reducing the dimensionality of large datasets containing highly correlated variables [54]. It identifies the underlying structure in a single set of variables by creating a smaller set of synthetic variables that explain most of the variability in the original data. Conversely to PCA, MFA allows for the analysis of multiple related data tables simultaneously. Individual PCA is conducted on each data table (table of continuous variables and table of categorical variables) to obtain the principal components and their associated variance. These individual PCA results are then combined using appropriate weighting schemes to create a joint factorial space. This joint space captures the overall relationships and variability between the different groups.

In the second step, the hierarchical clustering algorithm was applied to the principal component space [55]. The data points were grouped into phenogroups based on their proximity in the principal component space. The Euclidean distance, based on the concept of Euclidean geometry, where distances are measured along straight paths, was employed to determine the similarity between pairs of data points. This information was crucial for constructing the dendrogram, which visually represents the clustering process and enables the identification of phenogroups at different levels of similarity. Ward’s method was used to build the dendrogram tree.

The optimal number of phenogroups was suggested by the HCPC function itself, and the estimation was based on the between-variance. The optimal number of phenogroups was also estimated by using the NbClust function (Nbclust 3.0.1 package), which provided the calculation of 30 different indexes.

The over- or under-representation of variables in each phenogroup was evaluated by the v-test, based on the hypergeometric distribution, using the catdes function (FactoMineR 2.8 package).

### 2.8. Supervised Random Forest

Random forest (RF) is a machine learning algorithm classified as an ensemble method, comprising multiple decision trees trained on distinct subsets of data and features [56,57]. In the supervised setting, it necessitates labeled data with known target variables during the training phase.

In this study, the phenogroups generated by the unsupervised HCPC method were used as inputs for the supervised RF algorithm (randomForest function of the randomForest 4.7.1.1 package) to detect which patient parameters or features were most influential in distinguishing the phenogroups. The sample variables and number of trees were set to 4 and 500, respectively.

The mean decrease in the Gini coefficient was used to assess the importance of each input variable. A high mean decrease Gini score signifies higher relevance.

### 2.9. Statistical Analysis

All data were analyzed using SPSS version 27.0 (Chicago, IL, USA).

Continuous variables were presented as mean ± standard deviation (SD). Categorical variables were represented as counts with percentages.

The normality of the parameter distribution was evaluated through the Kolmogorov–Smirnov test.

Group comparisons were conducted using one-way ANOVA for continuous values with normal distribution and the Kruskal–Wallis test for continuous values with non-normal distributions. Χ^2^ testing was employed for categorical variables. The Bonferroni test was used for the post hoc analysis where appropriate.

The Cox proportional hazard model was employed to determine the effect of the phenogroups on the outcome. The results were presented as the hazard ratio (HR) with 95% confidence intervals (CI). Kaplan–Meier curves were generated and the log-rank test was employed to compare the different phenogroups regarding event-free survival.

In all tests, a 2-tailed probability value of 0.05 was considered statistically significant.

## 3. Results

### 3.1. Patients’ Characteristics

Twenty-three patients were excluded from the present study because a cardiac complication was present at the time of their first MRI. Specifically, seven patients had HF, six had arrhythmias, six had PH, two had HF and arrhythmias, and two had HF and PH.

Among the remaining 319 patients, 101 (31.7%) did not receive the contrast medium and were excluded from the analysis, reducing the study size to 218. SSFP images were not acquired for 13 patients because the lack of collaboration caused the advanced interruption of the exam. Moreover, out of the available 205 SSFP acquisitions, 67 were not collected in Pisa, making it not possible to assess the RV mass index. Therefore, the final study population comprised 138 patients for which all MRI parameters were measured.

Table 1 shows the clinical and MRI characteristics of the 138 considered patients. All patients were white and had a homogeneous sex distribution.

Replacement myocardial fibrosis was detected in 37 (26.8%) patients. Thirty-six patients had non-ischemic LGE patterns, while a subendocardial LGE pattern, typical of coronary artery disease, was found in one patient. Eleven patients had a single focus, while twenty-six had at least two foci. The mean number of LGE segments per patient was 2.4 ± 1.3. LGE involved the septal region in 81.1% of patients.

### 3.2. Clustering Results

The Hopkins statistic score was 0.99, revealing a strong clustering tendency within the set of observations.

The number of PCs to use as input for hierarchical clustering was chosen to guarantee at least 80% of the explained variance, and this was obtained with seven or more PCs. By choosing seven PCs, the dimension of the problem was strongly reduced: the sixteen original variables were replaced by seven synthetic variables (the seven PCs). Table 2 shows the percentage contributions of the variables to the seven dimensions.

Hierarchical clustering analysis indicated that the achievement of the highest gain in inertia or within-group variance was achieved with three phenogroups. Accordingly, the majority of indexes (18/30) evaluated by the Nbclust package proposed three as the best number of phenogroups for the considered dataset.

**Figure 1:**
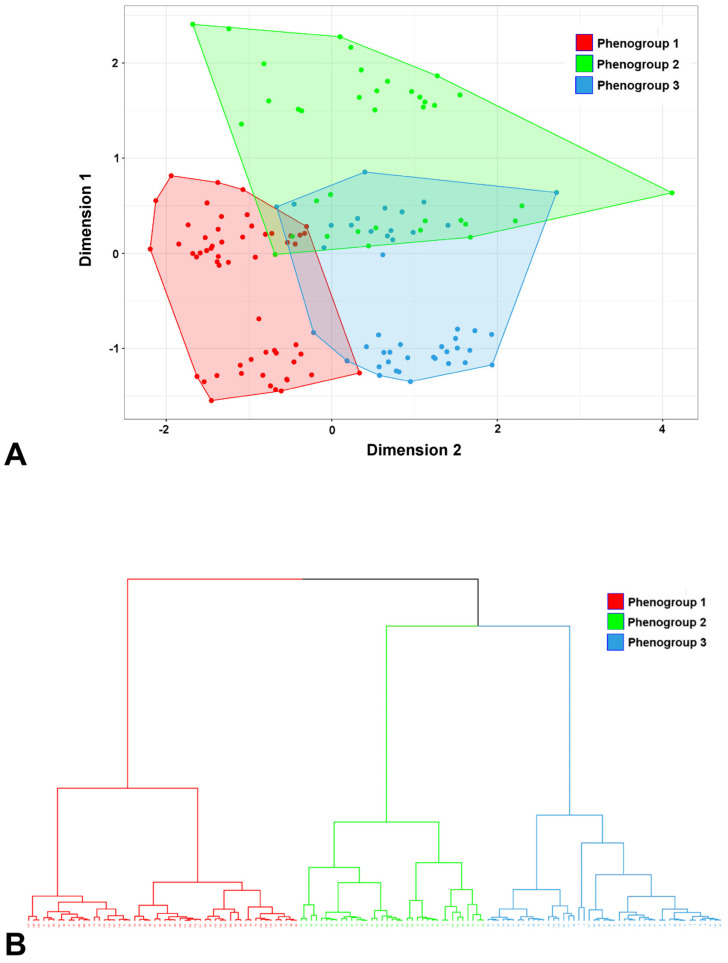
Cluster analysis. (**A**) Biplot with the first two components from the principal component analysis. Points represent individual patients displayed based on their individual characteristics. The colors represent the clear differentiation of the three phenogroups from the cluster analysis. (**B**) Dendrogram of the hierarchical cluster analysis. The ordinate axis represents the distance between merged phenogroups. Dashed lines indicate the partitioning with three phenogroups.

The three phenogroups are visible upon projection into a two-dimensional correspondence analysis biplot (Figure 1A). A dendrogram representation of the three phenogroups is presented in Figure 1B.

### 3.3. Comparison among Phenogroups

**Figure 2:**
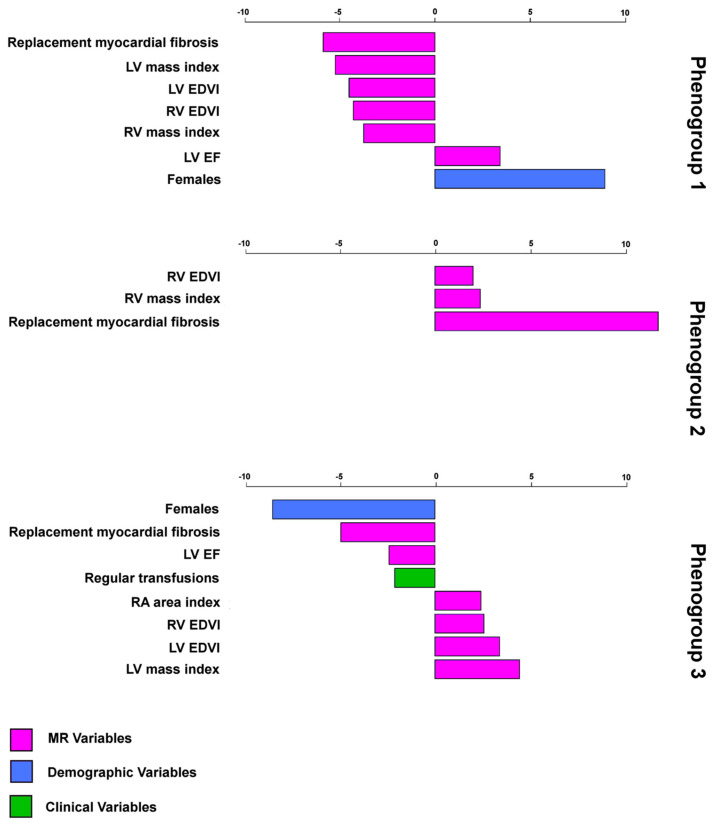
Characteristic plots of the three mutually exclusive phenogroups, including their most representative clinical variables. The over- or underrepresentation of a variable within a cluster was analyzed by a v-test within the hierarchical clustering of principal component function based on the hypergeometric distribution. A positive value indicates that the variable is overrepresented in the relevant phenogroup, while a negative value implies an underrepresentation of the corresponding variable. Only significant variables with *p* < 0.05 are listed.

Nine input variables contributed differently to the definition of the patient’s phenogroups (Figure 2). Age, hematochemical parameters, hepatic and cardiac iron levels, left trial area index, and RV EF did not contribute. Table 3 shows the differences among the three phenogroups.

Phenogroup 1 (56 patients; 40.6%) comprised almost exclusively women (98.2%). It exhibited significantly lower biventricular end-diastolic volume indexes and masses indexed by BSA and significantly higher LV EF compared with both phenogroups 2 and 3. No patient in this group had replacement myocardial fibrosis.

Phenogroup 2 (37 patients; 26.8%) was well balanced between sexes (45.9% females) and included all patients with replacement myocardial fibrosis.

Phenogroup 3 (45 patients; 32.6%) comprised only men and was characterized by the absence of replacement myocardial fibrosis.

### 3.4. Relevant Features for Clustering

**Figure 3:**
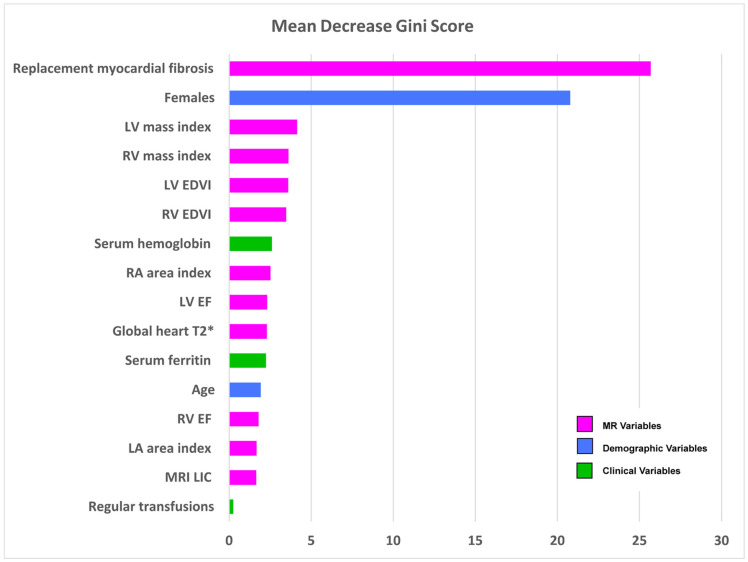
Variable importance plot from the supervised random forest model. Variables appear in decreasing order based on the contribution to the differentiation of patients in phenogroups.

In the supervised RF analysis, sex and replacement myocardial fibrosis by LGE emerged as the key parameters that significantly contributed to the differentiation of patients in each phenogroup (Figure 3). The presence of regular transfusions was associated with the lower mean Gini index.

### 3.5. Association of Phenogroups with Cardiovascular Complications

The mean follow-up time was 55.8 ± 22.7 months (median 54.0 months).

Cardiac events were recorded in 10 patients (7.2%). There was one episode of HF, five arrhythmias (all supraventricular), and four PH. The overall mean time elapsing between the first MR scan and the occurrence of a cardiac complication was 37.4 ± 26.5 months, and three (30.0%) cardiac events occurred within the first year of follow-up.

**Figure 4:**
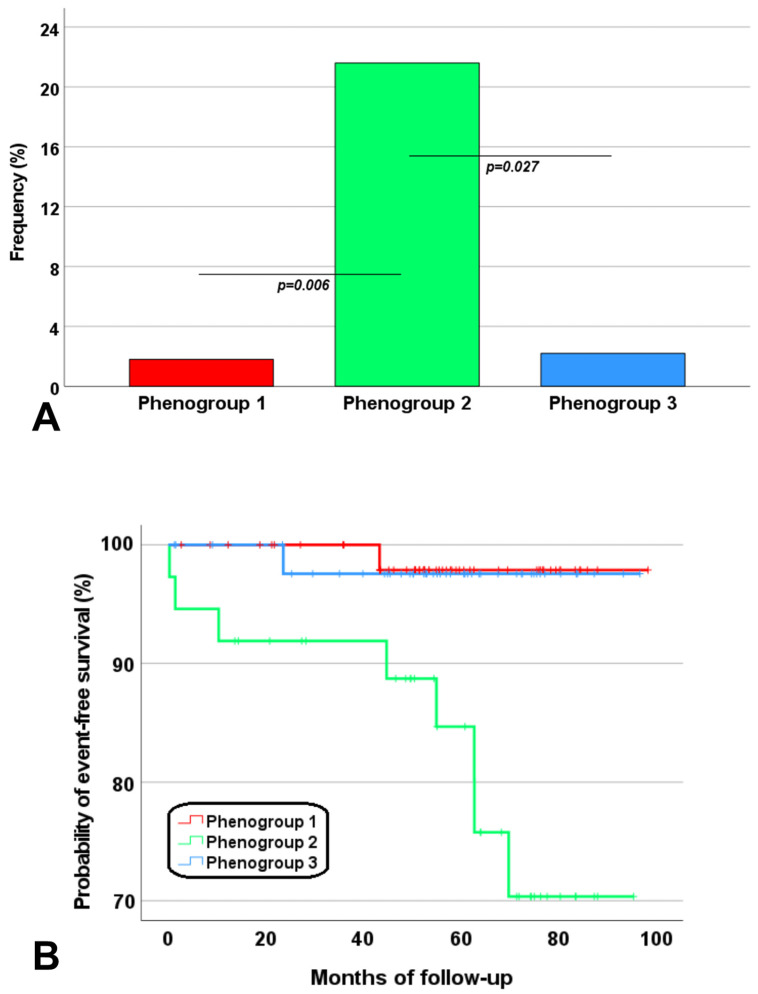
(**A**) Frequency of cardiovascular events in the three phenogroups. (**B**) Kaplan–Meier curves for cardiovascular events stratified by phenogroup.

The prevalence of cardiovascular events was significantly higher in phenogroup 2 than in both phenogroup 1 (21.6% vs. 1.8%; *p* = 0.006) and phenogroup 3 (21.6% vs. 2.2%; *p* = 0.027) (Figure 4A). Phenogroup 2 was associated with a significantly increased risk of cardiovascular complications compared with phenogroup 1 (HR = 10.5, 95%CI = 1.3–83.9; *p* = 0.027) as well as phenogroup 3 (HR = 9.0, 95%CI = 1.1–72.1; *p* = 0.038). Figure 4B shows the Kaplan–Meier curve. The log-rank test demonstrated significant differences in the occurrence of cardiovascular complications across phenogroups (*p* = 0.002).

## 4. Discussion

Machine learning algorithms possess the ability to process large volumes of data, identify patterns, and make predictions, which makes them well-suited for analyzing complex and diverse datasets commonly found in medical research. In the present study, we employed unsupervised clustering analysis on demographic, clinical, and CMR data with the aim of uncovering meaningful patterns and groupings within the β-TI population. Within the β-TI population, a substantial heterogeneity exists in patient characteristics, illness severity, and responses to treatment. Gaining a deeper insight into such heterogeneity could pave the way for more personalized care to better suit patient profiles. The statistical method of cluster analysis is a means by which such heterogeneity can be understood. We identified three mutually exclusive phenogroups of patients characterized by different levels of cardiac involvement and risks of cardiovascular outcomes.

Phenogroup 1 was primarily composed of female patients, while phenogroup 3 comprised only males. This gender difference may be the main cause of the difference between these two phenogroups in biventricular volumes and masses. Indeed, in healthy subjects [58,59] as well as in patients with hemoglobinopathies [50,60], all biventricular volume indexes as well as the LV mass index were demonstrated to be significantly greater in males compared with females. Conversely, phenogroup 1 was associated with a significantly higher LV EF. Several recent large-scale studies that used CMR or echocardiographic techniques detected a significantly greater LV EF in women compared with men [59,61,62]. Kerkhof et al. suggested that this finding may be explained by the disproportional increase in EF at smaller end-systolic volumes in women [63]. However, it is important to underline that phenogroups 1 and 3 were comparable in terms of prognosis. Therefore, although gender emerged as one of the key parameters that significantly contributed to the differentiation of patients in phenogroups, it did not play a significant role in cardiac risk stratification.

Phenogroup 2 included all patients with replacement myocardial fibrosis. These patients accounted for more than one-quarter of the total β-TI population (26.8%), indicating that replacement myocardial fibrosis is relatively common among Italian β-TI patients. Importantly, participants belonging to phenogroup 2 had a significantly increased risk of cardiovascular events compared with those in both phenogroups 1 and 3. The frequency of positive LGE was the only CMR parameter that significantly differed between phenogroups 2 and 3. At the same time, no difference was detected in biventricular volumes, ejection fractions, masses, or bi-atrial areas. These results suggest that, in β-TI, replacement myocardial fibrosis is potentially a better marker of cardiovascular risk stratification than conventional cardiac function parameters. In line with our findings, a growing body of evidence has shown the prognostic significance of LGE in predicting cardiovascular complications and death in both ischemic and nonischemic cardiomyopathies [64,65,66,67,68]. In particular, in thalassemia major, where the introduction of the T2* technique allowed for better management and control of cardiac iron, myocardial fibrosis emerged as the most potent predictor of HF and cardiac complications [69]. Unlike cardiac dysfunction and iron accumulation, fibrosis appears to be an irreversible process, suggestive of a potentially more significant and lasting injury to the myocardium. Although more studies involving larger cohorts of patients are needed to confirm the important prognostic implications of replacement myocardial fibrosis, the inclusion of LGE imaging within the cardiac MRI exam, if not contraindicated, can indeed be of significant value in the cardiological management of TI patients. The serial injection of Gadobutrol in patients with thalassemia without neurological lesions was demonstrated to be safe [51] and to not cause a signal hyperintensity of the dentate nucleus, globus pallidum, pons, and thalamus, potentially associated with the tissue deposition of the contrast media [70].

The cardiac T2* values did not provide sufficient discriminatory information to effectively distinguish or separate patients into their respective phenogroups. This result can be explained by the fact that the majority of our patients had normal (>20 ms) or only slightly reduced T2* values. It is well known that the T2* technique has reduced sensitivity for detecting changes associated with mild or early myocardial iron overload [71,72].

The three phenogroups were comparable in terms of the frequency of regular transfusions. This finding recalls a recent study that applied unsupervised RF clustering to identify subgroups of clinical severity in a large cohort of β-thalassemia patients, including TM patients, regularly transfused TI patients, and non-transfused TI patients [73]. Nineteen indicators of phenotype severity, which did not include MRI data, were considered, and the presence of regular transfusions did not play a significant role in grouping the patients into phenogroups.

### Limitations

Our study is not free from limitations.

The study was completed over 7 years ago and had a relatively small sample size. Moreover, all patients were from Italy, which possesses one of the best standards of care for thalassemia, arising from the high prevalence of the disease and availability of resources. All these factors may limit the generalizability of our findings. Therefore, new larger studies involving patients from different countries and backgrounds are needed to confirm our results.

As usual in clinical trials [74], complete case analysis (case deletion) was selected as the method for handling missing data in our dataset: all cases (patients) with missing data were discarded from the analysis. We basically assumed that the missing data were missing at random or completely at random (i.e., missingness was unrelated to the variables of interest or other observed characteristics). In this scenario, complete case analysis can yield unbiased estimates. Complete case analysis led to a significant decrease in the sample size, potentially reducing the statistical power of the analysis. Different approaches, like multiple imputation, maximum likelihood estimation, or weighted methods, can be used to impute or estimate the missing values [75]. Replacing missing clinical values with synthetically generated values enables the inclusion of all available cases in the analysis. However, imputing missing data involves making assumptions about the missing values, and these assumptions may not accurately represent the true values for the missing observations. In addition, imputation can alter the intrinsic characteristics of a dataset, introduce biases, and distort the relationships between variables.

Due to the limited number of events, we had to pool multiple types of cardiovascular events together, combining them into a single composite endpoint. Therefore, we could not evaluate the associations of the phenogroups with specific outcomes.

The current study was not designed to assess which CMR or clinical parameter was most powerful in predicting cardiovascular events.

The adopted approach represents a significant departure from traditional studies. Although phenogrouping holds the potential to pave the way for a more personalized approach to patient management, its actual impact on clinical decision-making is yet to be fully explored and understood.

## 5. Conclusions

Within the β-TI population, unsupervised learning algorithms, which integrated routinely measured CMR parameters, led to the identification of three phenogroups with distinct clinical and prognostic characteristics. Unsupervised phenogrouping conveys the potential to significantly impact patient care and improve cardiovascular outcomes by enabling the early detection of cardiac remodeling and damage, as well as enhanced risk stratification.

## Figures and Tables

**Table 1:** Demographic, clinical, and MRI data at the baseline MRI.

All β-TI Patients (N = 138)	
Females, N (%)	72 (52.2)
Age (years)	38.1 ± 11.4
Regular transfusions, N (%)	73 (52.9)
Serum hemoglobin (g/dL)	9.1 ± 1.1
Serum ferritin (ng/L)	813.8 ± 911.9
MRI LIC (mg/g/dw)	7.9 ± 7.7
Global heart T2* (ms)	35.9 ± 8.4
LV EDVI (mL/m^2^)	97.1 ± 20.6
LV mass index (g/m^2^)	63.6 ± 12.9
LV EF (%)	62.8 ± 5.9
RV EDVI (mL/m^2^)	90.0 ± 22.2
RV mass index (g/m^2^)	22.7 ± 6.2
RV EF (%)	65.3 ± 6.4
Replacement myocardial fibrosis, N(%)	37 (26.8)
LA area index (cm^2^/m^2^)	14.2 ± 2.6
RA area index (cm^2^/m^2^)	13.1 ± 2.5

β-TI = beta thalassemia intermedia, N = number, MRI = magnetic resonance imaging, LIC = liver iron concentration, LV = left ventricular, EDVI = end-diastolic volume index, EF = ejection fraction, RV = right ventricular, LA = left atrial, RA = right atrial.

**Table 2:** Contribution of continuous and categorical variables to the 7 dimensions. Only variables with a contribution > 5% are reported. Variables are ordered on the basis of the contribution level.

Dimension	Variance/ Cumulative Variance (%)	Variables with Contribution > 5%
1	22.3/22.3	Sex, LV EDVI, RV EDVI, LV mass index, RV mass index
2	16.5/38.8	Regular transfusions, replacement myocardial fibrosis
3	13.2/52.0	Replacement myocardial fibrosis, regular transfusions
4	11.4/63.4	Sex, RA area index, LA area index, RV EDVI, hemoglobin, LV EDVI, RV mass index
5	7.9/71.3	Ferritin, RV EF
6	6.9/78.2	Age, LV EF, RV EF, ferritin
7	4.6/82.8	Age, LA area index, RA area index, hemoglobin, RV EF, RV mass index

LV = left ventricular, EDVI = end-diastolic volume index, RV = right ventricular; RA = right atrial, LA = left atrial.

**Table 3:** Comparison of baseline demographic, clinical, and MRI findings among the three phenogroups.

	Phenogroup 1 (N = 56)	Phenogroup 2 (N = 37)	Phenogroup 3 (N = 45)	*p*-Value	Pairwise Comparisons
Females, N (%)	55 (98.2)	17 (45.9)	0 (0.0)	<0.0001	1 vs. 2: *p* < 0.0001 1 vs. 3: *p* < 0.0001 2 vs. 3: *p* < 0.0001
Age (years)	36.6 ± 12.3	39.8 ± 9.9	38.8 ± 11.5	0.380	
Regular transfusions, N (%)	34 (60.7)	21 (56.8)	18 (40.0)	0.100	
Serum hemoglobin (g/dL)	8.9 ± 0.8	9.0 ± 0.9	9.3 ± 1.4	0.349	
Serum ferritin (ng/L)	860.1 ± 956.1	977.9 ± 1142.9	621.1 ± 560.7	0.351	
MRI LIC (mg/g/dw)	8.5 ± 8.1	8.7 ± 8.4	6.5 ± 6.4	0.228	
Global heart T2* (ms)	36.3 ± 8.5	34.6 ± 10.6	36.6 ± 6.2	0.931	
LV EDVI (ml/m^2^)	87.6 ± 15.1	101.2 ± 20.8	105.7 ± 21.8	<0.0001	1 vs. 2: *p* = 0.003 1 vs. 3: *p* < 0.0001
LV mass index (g/m^2^)	56.7 ± 10.5	65.6 ± 12.2	70.6 ± 11.8	<0.0001	1 vs. 2: *p* = 0.001 1 vs. 3: *p* < 0.0001
LV EF (%)	64.9 ± 6.7	61.7 ± 4.9	61.0 ± 4.8	0.005	1 vs. 2: *p* = 0.045 1 vs. 3: *p* = 0.009
RV EDVI (mL/m^2^)	80.3 ± 17.2	96.3 ± 23.4	97.0 ± 22.6	<0.0001	1 vs. 2: *p* = 0.001 1 vs. 3: *p* < 0.0001
RV mass index (g/m^2^)	20.3 ± 3.8	24.7 ± 8.1	23.9 ± 6.0	<0.0001	1 vs. 2: *p* = 0.009 1 vs. 3: *p* < 0.0001
RV EF (%)	66.3 ± 7.1	63.9 ± 5.5	65.1 ± 6.2	0.201	
Replacement myocardial fibrosis, N (%)	0 (0.0)	37 (100.0)	0 (0.0)	<0.0001	1 vs. 2: *p* < 0.0001 2 vs. 3: *p* < 0.0001
LA area index (cm^2^/m^2^)	14.0 ± 2.6	14.3 ± 3.1	14.4 ± 2.3	0.765	
RA area index (cm^2^/m^2^)	12.7 ± 2.5	12.7 ± 2.5	13.8 ± 2.4	0.053	

N = number, MRI = magnetic resonance imaging, LIC = liver iron concentration, LV = left ventricular, EDVI = end-diastolic volume index, EF = ejection fraction, RV = right ventricular, LA = left atrial, RA = right atrial.

## Data Availability

The data underlying this article cannot be shared publicly due to privacy reasons. The data will be shared on reasonable request to the corresponding author.
